# Elementary Flux Mode Analysis of Acetyl-CoA Pathway in *Carboxydothermus hydrogenoformans* Z-2901

**DOI:** 10.1155/2014/928038

**Published:** 2014-04-16

**Authors:** Rajadurai Chinnasamy Perumal, Ashok Selvaraj, Gopal Ramesh Kumar

**Affiliations:** Bioinformatics Lab, AU-KBC Research Centre, M.I.T Campus of Anna University, Chromepet, Chennai, Tamil Nadu 600 044, India

## Abstract

*Carboxydothermus hydrogenoformans* is a carboxydotrophic hydrogenogenic bacterium species that produces hydrogen molecule by utilizing carbon monoxide (CO) or pyruvate as a carbon source. To investigate the underlying biochemical mechanism of hydrogen production, an elementary mode analysis of acetyl-CoA pathway was performed to determine the intermediate fluxes by combining linear programming (LP) method available in CellNetAnalyzer software. We hypothesized that addition of enzymes necessary for carbon monoxide fixation and pyruvate dissimilation would enhance the theoretical yield of hydrogen. An *in silico* gene knockout of *pyk*, *pykC*, and *mdh* genes of modeled acetyl-CoA pathway allows the maximum theoretical hydrogen yield of 47.62 mmol/gCDW/h for 1 mole of carbon monoxide (CO) uptake. The obtained hydrogen yield is comparatively two times greater than the previous experimental data. Therefore, it could be concluded that this elementary flux mode analysis is a crucial way to achieve efficient hydrogen production through acetyl-CoA pathway and act as a model for strain improvement.

## 1. Introduction

Use of fossil fuels causes adverse effect on environment through pollution. Moreover, the availability of fuels such as oils and natural gases is limited and are likely to be depleted soon [1]. Therefore, it is indispensable to search for alternate fuel source and hydrogen is one of the efficient sources of energy that could effectively replace the available fossil fuels. It is also considered as fuel of the future, since it is eco-friendly and emits zero carbon. Besides its application as a fuel, hydrogen can also be used as a potential electron donor for various reactions in biotechnological and chemical industrial processes [2]. Hydrogen is conventionally produced from fossil fuels by steam reforming other industrial methods such as coal gasification and electrolysis [3]. However, these methods uses nonrenewable energy source to produce hydrogen. Therefore, biological hydrogen production by microorganisms especially by hydrogenogens is the most convenient one [4, 5]. Over the past two decades, various researches are going on for enhanced biological hydrogen productivity [6, 7] and improvement of such a product from organisms by optimizing their genetic process is commonly referred to as metabolic engineering [8]. The knowledge of reactions and selection of optimal enzymatic route between the substrate and product is an ultimate task in biological research. Computational based theoretical metabolic yield is a key criterion to study the substrate utilization and product formation of microorganisms [9–11].* Carboxydothermus hydrogenoformans* Z-2901 is one of the most promising and potential acetogenic hydrogenogenic bacterium produces species which produces hydrogen by utilizing CO and pyruvate as a carbon source [12]. In hydrogenogenic microorganisms, the autotrophic fixation of CO and pyruvate dissimilation have been achieved through acetyl-CoA or Wood-Ljungdahl pathway and hydrogen molecule has been produced as one of the end product. The reactions involved in CO fixation are activated into acetyl group that contains metal ions. The* CooX* cluster of genes is the major component of this pathway that fixes CO and pyruvate dissimilation catalyzed by* pdh* gene during acetyl-CoA pathway, a key biochemical feature that supports hydrogen production [13, 14].

Elementary flux mode (EFM) analysis is one of the powerful tools for metabolic pathway analysis. It allows us to calculate all possible steady-state flux distributions of the network, thereby determining the theoretical molar yield of products and studying their effects of any genetic modifications [15–17]. Such studies would help to design an organism for obtaining the efficient product formation through metabolic engineering. Recently, elementary mode analysis has been used to develop a rational model of methionine production from well-known organisms such as* Escherichia coli *and* Corynebacterium glutamicum* [18], and polyhydroxybutanoate production from* Saccharomyces cerevisiae* [19–21]. The experimental analysis of hydrogen production in wild type* E. coli* resulted in a flux distribution indicating a hydrogen production of 0.17 mol per mole of glucose consumption [22]. The predicted hydrogen production of* E. coli* through metabolic flux analysis by the deletion of* ldh* gene was 0.23 mol per mole of glucose consumed which is slightly higher than the wild type strain. In another case, computational flux analysis studies on hydrogen production in* E. coli* through the formate hydrogen lyase reaction have suggested that the level of hydrogen production matches experimental observations [23]. In this paper, elementary mode based flux analysis has been carried out for the newly modeled acetyl-CoA pathway of* C. hydrogenoformans* that comprises necessary reaction stoichiometry collected from KEGG database [24]. Theoretical capabilities of hydrogen yield limited by the utilization of CO and pyruvate and through* in silico *gene knockout have been studied using metabolic flux analysis tools and software. This elementary mode flux analysis also provides a basis to design a system that has specific phenotypes, metabolic network regulation, and robustness that facilitates the understanding of cell physiology and implementation of metabolic engineering strategies of* C. hydrogenoformans* to improve the hydrogen productivity.

## 2. Materials and Methods

### 2.1. Reconstruction and Modeling of Acetyl-CoA Pathway

A simplified model of acetyl-CoA pathway of* C. hydrogenoformans* was constructed based on biochemical features consisting of stoichiometry reactions retrieved from KEGG (Kyoto Encyclopedia of Genes and Genome) database [24]. The KEGG database was used to link annotated genes to protein and to reactions. Gaps in acetyl-CoA pathway were filled based on homology search and context analysis using KEGG-BLAST against orthologs [25, 26]. Initially, the protein ORF sequences (both known and unknown ORFs) of* C. hydrogenoformans* were submitted to KEGG-BLAST tool to find out the reaction information of functionally characterized orthologous gene hits. It was manually checked that the product of orthologous reaction hits should match with reactant of reaction present in the same or any of the pathways of* C. hydrogenoformans*. The metabolite (both substrate and product) of all the unknown reactions has been interconnected to make a subnetwork using Cytoscape software [27]. A cytosolic compartment was applied to ideally depict the influx and efflux rate of substrate, cofactors, and product [28]. This set of modeled reaction equations was further used for elementary flux mode analysis to predict the theoretical yield of hydrogen and other products. The modeled acetyl-CoA pathway includes both pyruvate dissimilation and methane degradation metabolism [29–31]. For the interconversion of CO to CO_2_ during acetyl-CoA pathway_,_ CO dehydrogenase enzyme and pyruvate dehydrogenase activities were initially considered. The precursors such as NADP and ATP necessary for product formation were also included in the reaction stoichiometry. With the use of collected reaction data as discussed above, flux balance-based models of pyruvate and methane metabolism have been built and are known as acetyl-CoA carbon metabolism or Wood-Ljungdahl pathway of* C. hydrogenoformans* [32].

### 2.2. Elementary Flux Mode Analysis

The elementary flux mode analysis was carried out for studying the biochemical behavior of acetyl-CoA pathway of* C. hydrogenoformans*. The stoichiometric reactions of modeled acetyl-CoA pathway were imported and analyzed using CellNetAnalyzer with MATLAB [32]. The METATOOL program available in CellNetAnalyzer was compiled using MATLAB environment to generate elementary modes having specific reaction sets. The objectives of reactions that maximize the flux rate of products have been calculated using linear programming (LP). For flux optimizations, a GLPK library, GNU based linear programming kit was used through the GLPKMEX interface. The theoretical fluxes were calculated for the substrates pyruvate, CO, and other by-products such as hydrogen, malate, oxaloacetate, and formate.

During metabolic flux analysis, the objective is usually maximizing the flux rate of products or biomass [33]. Here, we considered pyruvate and CO influxes as objective for maximizing the hydrogen and other product formation. The theoretical molar yield of any product is represented by [*vp*/*vs*], where *vp* is flux rate of product and *vs* is flux rate of substrate. The increase of product yield can be achieved by maximizing the flux *vp*, constrained by the flux *vs* of the substrate. The flux balance analysis is described in a mathematical formulation by the following equation:

(1) $$\begin{array}{c} \overset{}{\underset{j}{\sum }}s_{ij}v_{j}=0,\;\forall i\in M_{i}, \end{array}$$

where *S*_*ij*_ is the stoichiometric coefficient of the *i*th metabolite in the *j*th reaction and *V*_*j*_ is the flux of the *j*th reaction. *M*_*i*_ is the internal metabolites. The flux distributions of the metabolic network model in steady state expressed by (1) was solved using METATOOL software [34].

## 3. Results 

### 3.1. Identification of Essential Reactions for Elementary Flux Mode Studies

**Figure 1:**
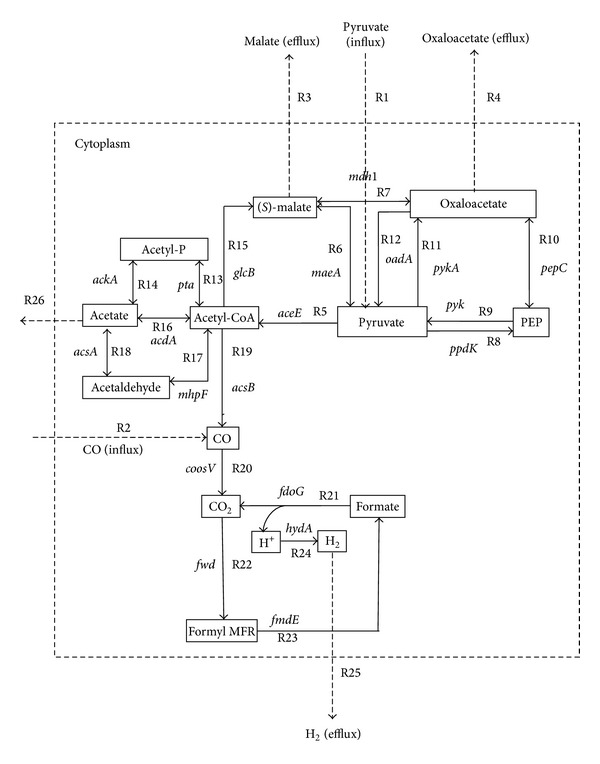
The modeled acetyl-CoA pathway of* C. hydrogenoformans* utilizing CO and pyruvate as a carbon source. Black color line shows that the reactions obtained from topology of KEGG orthologous pathway map. The influx and efflux directions of substrates and products were indicated as dotted lines. The Rn indicates the reaction numbers in Table 1.

To identify the essentiality of ORFs that code for energy producing enzymes and cofactors, an* in silico *gene knockout studies hace been performed [35]. With the use of metabolic stoichiometry data available from KEGG database, a flux balance-based model of pyruvate and methane metabolism of* C. hydrogenoformans* has been built and it is illustrated in Figure 1. This constructed model can be utilized for industrial based hydrogen production. Totally, twenty- two reactions were collected from KEGG database (excluding influx and efflux reactions) (Table 1) and they were involved in reducing acetyl-CoA during methane metabolism (KEGG Pathway ID: map00680). Here, the flux rate of the newly added enzymes which influence the hydrogen production was identified by* in silico* gene deletion method using CellNetAnalyzer software with MATLAB [32]. The initial uptake of substrate and product formation rates is essential for the enhancement of the growth of* C. hydrogenoformans*.

### 3.2. Identification of Elementary Modes

**Figure 2:**
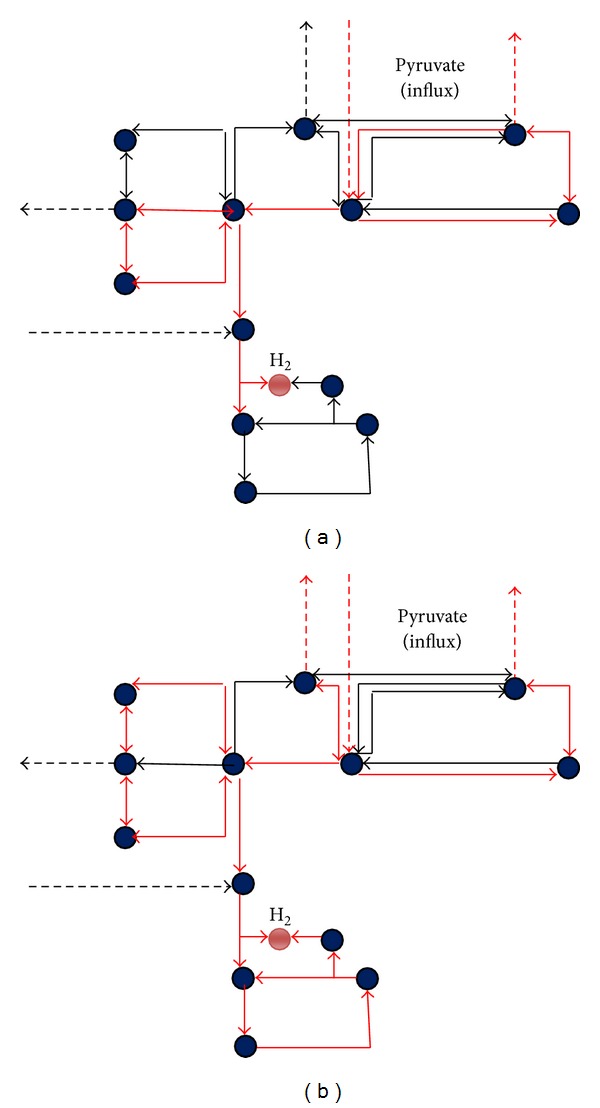
Important elementary modes of acetyl-CoA pathway of* C. hydrogenoformans*. (a) Elementary mode 9 consists of minimal reaction sets required for hydrogen production; (b) elementary mode 21 of acetyl-CoA metabolism during which maximum hydrogen was obtained.

Elementary modes (EMs) are the minimal sets of reactions catalyzed by enzymes that allow the network to perform at steady state [36, 37]. They represent the optimal route of utilizing external substrate and forming external products and thus they are defined in the context of whole-cell metabolism. The modeled acetyl-CoA metabolic pathway was taken for this elementary mode flux analysis studies. In this pathway, carbon monoxide (CO) and pyruvate are the substrate dissimilates to form acetyl-CoA as an important intermediate and yielding malate, acetate, oxaloacetate, and hydrogen as end products. This pathway is also called as Wood-Ljungdahl pathway, since it utilizes CO as a substrate catalyzed by CO dehydrogenase enzyme and converted to acetyl-CoA [38]. In this modeled pathway, the number of metabolites are lesser than number of reactions and therefore satisfies under-deterministic condition [37]. The resulting network comprises totally 26 reactions with 18 metabolites (Table 1). About 8 reactions are reversible and 18 reactions are irreversible. As a result of elementary mode analysis, about 28 elementary modes (EMs) were derived from this pathway totally and among them 18 EMs lead to the formation of hydrogen from pyruvate and CO. Those 18 EMs can be further classified according to their gene catalyzed and product formation. Elementary mode distributions of major metabolites are malate 16 EMs, oxaloacetate 14 EMs, acetate 4 Ems, and hydrogen 18 EMs. The rest of EMs tends to form other compounds such as malate, oxaloacetate, and acetate. Two elementary modes EM 9 and EM 21 are predicted to be having minimal reaction sets for hydrogen production by consumption of pyruvate as a substrate (Figure 2). They attained the steady-state level after allowing the substrate into the system. During acetyl-CoA pathway, utilization of CO by* C. hydrogenoformans* is catalyzed by two major enzymes, CO dehydrogenase and acetyl-CoA synthase [32]. The influx rate of CO and/or pyruvate (substrate) determines the intermediate carbon flux, efflux rate of hydrogen, and other metabolites.

### 3.3. Gene Participation in EMs

**Figure 3:**
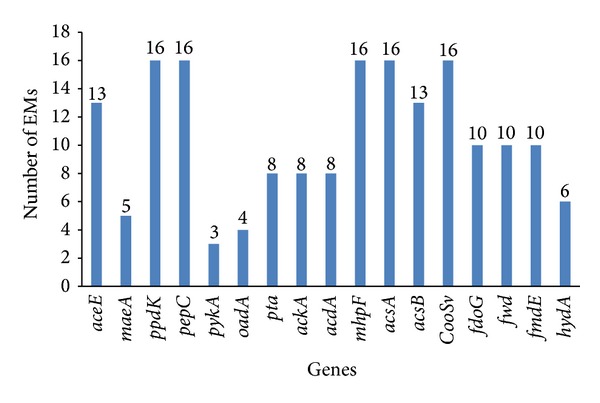
Types of gene involved in hydrogen production during acetyl-CoA pathway of* C. hydrogenoformans*. The genes* ppdK, pepC, mhpF, acsA,* and* CooSv* are predicted to be involved in all the eighteen elementary modes.

The occurrence of genes involved in hydrogen production during acetyl-CoA pathway was studied through elementary mode analysis.  Figure 3 shows that there are seventeen genes that appear in eighteen elementary modes which are the optimal routes responsible for synthesizing hydrogen. Such modes can be characterized by indicating genes sets or enzymes involved in the product formation. The gene sets* ppdK, pepC, mhpF, acsA,* and* CooSV* catalyze reactions with pyruvate and/or CO as carbon sources and they participated in all the eighteen elementary modes (EMs) in acetyl-CoA pathway. The genes* aceA* and* acsB* furthest down the pathway participate in only thirteen EMs. Next to this, genes such as* fdoG, fwd, *and* fmdE *were involved in only ten EMs and genes* maeA, pykA, oadA, hydD, pta, ackA, *and* acdA* participated in less than ten EMs. These findings suggested that the blocking of those gene sets or the reactions does not affect carbon flow during acetyl-CoA pathway. The genes* acs* and* pyk* are involved in many modes, which are predicted to have major role for maintaining the steady-state flux throughout the pathway.

Malate is one of the important metabolites formed from pyruvate during CO metabolism. Oxaloacetate and acetate are other important metabolites formed by the decarboxylation of pyruvate [39]. Thus, if the genes related to hydrogen will be removed, the formation of malate, oxaloacetate, and acetate will be affected. These discrepancies could cause severe physiological change of the organism.

### 3.4. Elementary Flux Mode Analysis of Acetyl-CoA Pathway

Elementary flux mode analysis of acetyl-CoA pathway helps to predict the promising gene deletion target necessary for increasing the product rate [40]. This is the first reported elementary flux mode analysis result of* C. hydrogenoformans* for the purpose of augmentation of hydrogen production. By examining the elementary modes (EMs) of acetyl-CoA pathway, the key reaction steps for efficient hydrogen production have been identified by carrying out flux optimization using METATOOL option available in CellNetAnalyzer software [32]. As a result of flux optimization, the theoretical molar yield of hydrogen has been calculated with different substrate and product combination. It was assumed that the flux rate and concentration of malate, oxaloacetate, acetate, and formate determine the cellular growth of CO oxidizing bacterial species that include carboxydotrophic hydrogenogens [41, 42]. The level of ATP and NADP (co-factors) were also calculated during elementary flux mode analysis.

By generating stoichiometric matrix, the metabolite connections between reactions can be studied to identify the feasible steady-state metabolic flow. These EM studies help to investigate the important subnetworks, their gene participation, and their functionalities for obtaining hydrogen and other important metabolites through acetyl-CoA pathway. This study also help in identifying genes, when deleted through gene knockout would increase hydrogen production. For maintaining the robustness and its sensitivity to perturbation such as new reaction inclusion, gene knockouts were completely analyzed to optimize the modeled acetyl-CoA pathway. The sensitivity of hydrogen yield to the flux values of pyruvate, CO utilization, and acetyl-CoA formation was studied in this work.

### 3.5. *In Silico* Gene Knockout Analysis

The gene deletion or knockout studies could have either positive or negative effects on phenotypes of organisms. Essential genes and reactions in the EFM pathways could not be deleted [43]. For the model of acetyl-CoA pathway, elementary mode (EM) has been systematically used to examine gene deletion that influence the hydrogen yield of* C. hydrogenoformans* as well as to identify a set of multiple gene knockout which results in higher product yield. Gene knockouts were carried out by removing the enzymatic reaction corresponding to that gene (having zero constraints) from the stoichiometric matrix. Totally, 28 elementary modes were observed and their fluxes were measured. Among them, only two EMs 9 and 21 gene sets or reaction flow have maintained the maximum possible yield of hydrogen and retain a reasonable yield of of by-products such as acetate, malate, and oxaloacetate while the other possible number of elementary modes with minimal product fluxes was eliminated.

**Figure 4:**
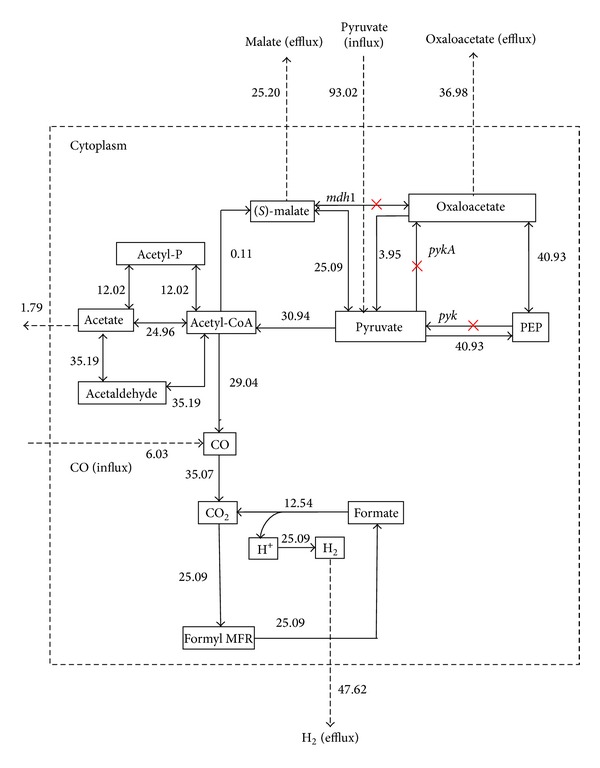
Gene knockout on elementary mode 21 of acetyl-CoA pathway in* C. hydrogenoformans*. The obtained fluxes were highlighted in blue color box. Knockout genes* pyk, pykA,* and* mdh* genes were highlighted in red mark (×).

**Figure 5:**
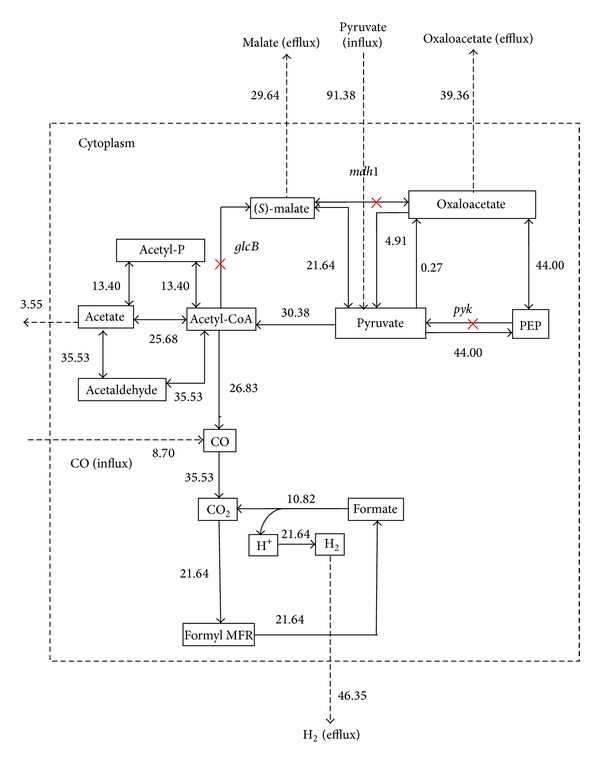
Gene knockout on elementary mode 9 of acetyl-CoA pathway in* C. hydrogenoformans*. The knockout genes* glcB, mdh,* and* pyk* were highlighted in red mark (×). Here, the reactions connecting the malate and oxaloacetate were blocked and fluxes were maintained in alternate nodes.

**Figure 6:**
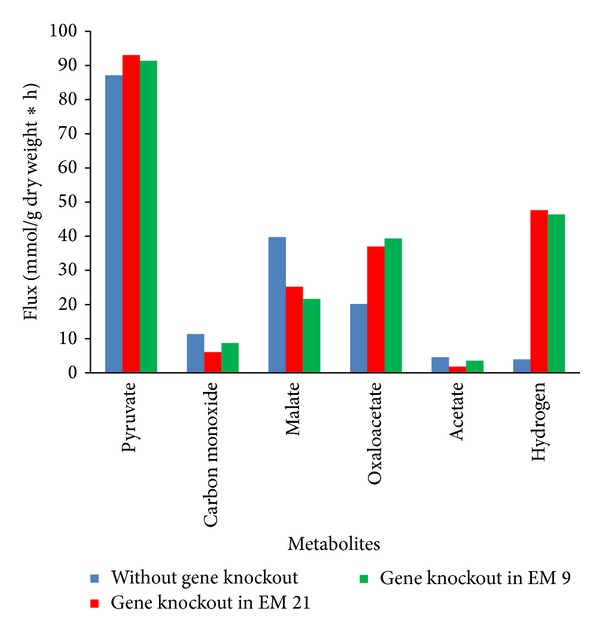
The fluxes of hydrogen and other major metabolites during acetyl-CoA pathway of* C. hydrogenoformans.* (A) Blue bar represents the flux rates of metabolites without gene knockout. (B) Red bar shows the maximum fluxes obtained from elementary mode 21 by gene knockout of* pyk, pykA,* and* mdh* genes. (C) Green bar represents the flux rates obtained from elementary mode 9 by gene knockout of* glcB, mdh,* and* pyk* genes.

For metabolic flux analysis study, we used the modeled acetyl-CoA pathway (Figure 1) to achieve the enhanced theoretical molar yield of hydrogen. Three genes* pyk, pykA,* and* mdh* were knocked out during flux optimization of EM 21 (Table 2). A cytosolic compartment has been included in network model to measure the external fluxes such as substrate uptake and product formation. As a result, the influx or consumption rate of pyruvate reaches from 87.11 mmol/gCDW/h to 93.02 mmol/gCDW/h and thereby increases the hydrogen flux from 38.95 mmol/gCDW/h to 47.62 mmol/gCDW/h (Figure 4). The increases in the flux value actually indicate fixation of CO by* CooS *gene and decarboxylation of pyruvate by* pyk *gene [32, 44]. During the gene knockout study on EM 9, malate synthase (*glcB*), malate dehydrogenase (*mdh*), and pyruvate kinase (*pyk*) genes were knocked out (Figure 5, Table 2) and, as a result, the flux rate of hydrogen reached to 46.35 mmol/gCDW/h. The influence of flux rate of other byproducts such as malate, oxaloacetate and acetate on hydrogen production were also examined. The flux distribution of pyruvate, CO, hydrogen, and other major metabolites during normal flux measurement (i.e) without gene knockout is clearly illustrated in Figure 6(A). As a result, flux rate of hydrogen reaches 38.95 mmol/gCDW/h. Maximum flux of 47.62 mmol/gCDW/h of hydrogen was obtained in the twenty-one elementary mode and it is clearly elucidated in Figure 6(B). This flux rate is slightly higher than the normal flux (i.e) flux obtained without gene knockout (Figure 6(A)). During this mode, only pyruvate was influxed forming hydrogen and malate as a product. The enzymes such as pyruvate kinase (pyk), pyruvate carboxylase (pykA), and malate dehydrogenase (mdh) have been knocked out since they are not involved in EM 21 (Table 2). The other major products obtained during EM 21 were malate, oxaloacetate, and acetate. Similarly, the EM 9 has the maximum flux rate of hydrogen formation next to the level of EM 21. The obtained flux rates of hydrogen and other metabolites in EM 9 were illustrated in Figure 6(C).

## 4. Discussion

*In C. hydrogenoformans, *pyruvate is initially converted to acetyl-CoA and formate by pyruvate formate lyase (pfl) enzyme and formate are subsequently metabolized into hydrogen and carbon dioxide. In another case, CO can be directly fixed and converted into CO_2_ and hydrogen through water gas shift reaction and this reaction is being catalyzed by CO dehydrogenase enzyme complex [45]. Our strategy for improving hydrogen production involved the modification of energy metabolism to direct the flow of major metabolites pyruvate and acetyl-CoA through elementary flux mode analysis. The biochemical reactions comprised in acetyl-CoA pathway are illustrated in Figure 1 and the functions of corresponding genes are summarized in Table 1. This is the first flux analysis study reported on acetyl-CoA pathway and hydrogen production of* C. hydrogenoformans*. The gene knockouts of selected genes (Table 2) obtained from elementary mode analysis were hypothesized to increase the pyruvate and CO influx rate, thereby increasing the flux rate of hydrogen production. The rate of* pepc *and* pyc *genes encoding phosphoenolpyruvate (PEP) carboxylase and pyruvate kinase was disrupted to increase the pyruvate concentration in cellular system for acetyl-CoA synthesis [39, 46]. A previous metabolic engineering study by gene deletion on model microorganism* Escherichia coli* described that one mole of glucose has maximum hydrogen yields of approximately 14.9 mmols/mg dry cell mass [47]. Similarly, the overexpression studies on transcriptional regulatory genes,* fhl* and* fnr* genes of* E. coli,* resulted in the yield of 34 mmol of H_2_/mg of dry cell mass [48]. As a result of elementary mode flux analysis, we obtained a maximum theoretical flux rate of hydrogen yield of 47.62 mmol/gCDW/h for one mole of pyruvate consumption was comparatively comparatively higher than* E. coli* simulation. The elimination of* fdh* and* pyk *genes in* E. coli* strains also results in increase of pyruvate metabolism towards hydrogen [49]. Here, we suggested that gene knockout of* pyk* and* mdh* genes would increase the flux rate of hydrogen during pyruvate dissimilation of acetyl-CoA pathway in* C. hydrogenoformans*. Thus, the analysis of the elementary mode based fluxes showed that the hydrogen yield through acetyl-CoA pathway model proposed here was validated with the experimental data. As acetyl-CoA is one of the precursors of hydrogen formation and the yield depends on the rate of acetyl-CoA and other intermediate produced [50], it was keenly observed that* pyk* and* mdh* genes involved in both EMs 9 and 21 were obtained zero constraints, since their absence does not affect the carbon flux during acetyl-CoA pathway. This* in silico* elementary mode analysis and flux analysis of acetyl-CoA model indicated that the reactions available are feasible for the carbon flow from substrates pyruvate and CO to produce maximum amount of hydrogen. After flux optimization, EMs 21 and 9 (Figures 2(a) and 2(b)) were predicted to be the efficient reaction sets for enhanced hydrogen productivity. As discussed above, to improve the hydrogen yield by* C. hydrogenoformans*, a gene knockout of* mdh*,* pyk,* and* pykA* genes during acetyl-CoA pathway would be a prior consideration. Gene knockout of* pyk* and* pykA* could redistribute the flux of pyruvate into acetyl-CoA synthesis for hydrogen production. It has been reported that knock-out of pyruvate kinase (pyk), pyruvate kinase type-II (pykA), and malate dehydrogenase (mdh) enzymes could increase both the growth rate and yield of hydrogen [11, 39, 50]. The knockout of enzyme pyruvate kinase controls the flux from PEP towards pyruvate, resulting in a relative difference in the rate of carbon flow toward oxaloacetate and other products such as formate and acetate [39]. Our results clearly showed that the disruption of pyruvate kinase enzyme activity maintains the steady-state flux through the combined reactions of oxaloacetate to pyruvate and pyruvate to phosphoenolpyruvate. The results presented in this work illustrate the fixation of CO, dissimilation of pyruvate, and formate related to acetyl-CoA biosynthesis, to achieve the maximum theoretical hydrogen yield. Thus, undercontrolled intake of pyruvate, metabolic perturbations, resulting from pyruvate kinase, and malate dehydrogenase gene knockout led to strongly increase the flux rate of hydrogen formation. proposed* in silico* gene knockout and flux analysis model in this paper will help to improve the strain through metabolic engineering for obtaining the enhanced hydrogen production phenotype.

## Figures and Tables

**Table 1:** Reactions and enzymes involved in the acetyl-CoA pathway model of *C. hydrogenoformans*.

Reaction number	Reactions	Gene name	EC number	Enzyme
R 1:	pyruvate intake	∗	∗	Influx
R 2:	*⟹* CO	∗	∗	Influx
R 3:	malate *⟹*	#	#	Efflux
R 4:	OAA *⟹*	#	#	Efflux
R 5:	pyr *⟹* CO2 + ACoA	*aceE *	1.2.4.1	Pyruvate dehydrogenase E1
R 6:	pyr + CO2 *⟺* malate	*maeA *	1.1.1.38	Malic enzyme
R 7:	OAA + NADH *⟺* malate	*mdh1 *	1.1.1.37	Malate dehydrogenase
R 8:	pyr + ATP *⟹* PEP	*ppdK *	2.7.9.1	Pyruvate phosphate dikinase
R 9:	PEP *⟹* pyr + ATP	*pyk *	2.7.1.40	Pyruvate kinase
R 10:	CO2 + PEP *⟺* OAA	*pepC *	4.1.1.31	PEP carboxylase
R 11:	pyr + CO2 *⟹* OAA	*pykA *	6.4.1.1	Pyruvate kinase II
R 12:	OAA *⟹* pyr + ATP	*oadA *	4.1.1.3	Pyruvate carboxylase subunit
R 13:	ACoA *⟺* acetylP	*pta *	2.3.1.8	Phosphate acetyltransferase
R 14:	acetylP *⟺* ATP + acetate	*ackA *	2.7.2.1	Acetate kinase
R 15:	ACoA + H2O *⟹* malate	*glcB *	2.3.3.9	Malate synthase G
R 16:	ACoA *⟺* ATP + acetate	*acdA *	6.2.1.13	Acetyl-CoA synthase
R 17:	acetaldehyde *⟺* ACoA	*mhpF *	1.2.1.10	Acetaldehyde dehydrogenase
R 18:	NADH + acetate *⟺* H2O + acetaldehyde	*acsA *	6.2.1.1	Acetyl-coenzyme A synthetase
R 19:	ACoA *⟹* CO	*acsB *	**2.3.1.169**	CO-methylating acetyl-CoA synthase
R 20:	H2O + CO *⟹* CO2 + H2	*cooSV *	1.2.99.2	Carbon-monoxide dehydrogenase
R 21:	Formate + NAD^+^ *⟹* CO2 + NADH	*fdoG *	1.2.1.2	Formate dehydrogenase-O
R 22:	CO2 + MFR *⟹* H2O + fMFR	*fwd *	1.2.99.5	Tungsten formylmethanofuran dehydrogenase subunit E
R 23:	fMFR + H2O *⟹* Formate + MFR	*fmdE *	1.2.99.5	Formylmethanofuran dehydrogenase
R 24:	2 H^+^ *⟹* H2	*hydA *	1.12.7.2	Ni/Fe hydrogenase large subunit
R 25:	H2 *⟹*	#	#	Efflux
R 26:	Acetate *⟹*	#	#	Efflux

*Substrate influx into the system.

^#^External product efflux from the system.

**Table 2:** Knockout genes in elementary mode 21 and elementary mode 9.

Reaction number	EC number	Enzyme name	Knockout gene name
Knockout genes in elementary mode 21			
R 9	2.7.1.40	Pyruvate kinase	*pyk*
R 11	6.4.1.1	Pyruvate carboxylase	*pykA*
R 7	1.1.1.37	Malate dehydrogenase	*mdh*

Knockout genes in elementary mode 9			
R 15	2.3.3.9	Malate synthase	*glcB*
R 7	1.1.37	Malate dehydrogenase	*mdh*
R 9	2.7.1.40	Pyruvate kinase	*pyk*
